# Novel microwave surgical instrument for use in various lung resection situations

**DOI:** 10.1186/s13019-021-01692-8

**Published:** 2021-10-18

**Authors:** Atsushi Kamigaichi, Takeshi Mimura, Yoshinori Yamashita

**Affiliations:** Department of General Thoracic Surgery, National Hospital Organization Kure Medical Center and Chugoku Cancer Center, 3-1, Aoyama-cho, Kure, Hiroshima 737-0023 Japan

**Keywords:** Microwave surgical instrument, Incomplete interlobar fissure, Segmentectomy, Wedge resection

## Abstract

A novel surgical energy device with high sealing ability using microwave technology has been developed. This novel microwave surgical instrument (MSI) is capable of sealing and dissecting a vessel ≤ 5 mm in diameter. The high sealing ability of the MSI enables fine dissection of the lung parenchyma by a scissor-type blade. This device is particularly useful in situations wherein the use of an automatic suturing instrument is difficult. Here, we describe the dissection of the lung parenchyma using this device in three patients (cases 1–3). This device was used for wedge resection of a tumor located close to the pulmonary hilum, for subsegmentectomy, and for dividing incomplete interlobar fissure (cases 1–3, respectively). In all the cases, the postoperative course was uneventful. This MSI is effective for resection of the lung parenchyma, allowing fine tissue dissection and excellent tissue sealing. This technique could assist surgeons in various lung resection cases.

**Supplementary Information:**

The online version contains supplementary material available at 10.1186/s13019-021-01692-8.

## Introduction

**Fig. 1:**
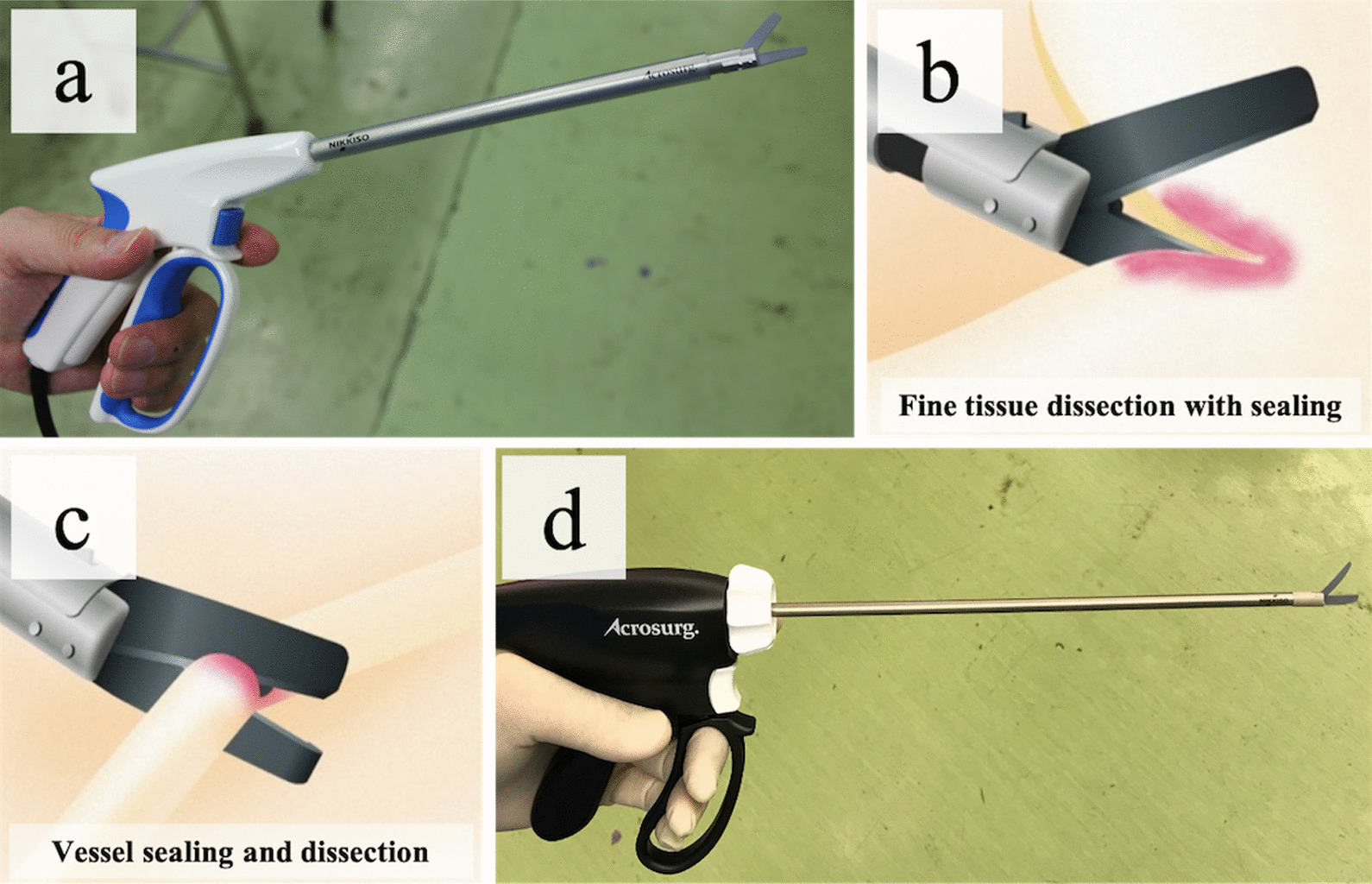
**(a)** Acrosurg.® (Nikkiso Co Ltd, Tokyo, Japan) with a short shaft length of 17 cm. The microwave surgical instrument allows fine tissue dissection (**b**) and vessel sealing (**c**). (**d)** Acrosurg.® Revo (Nikkiso) with a longer shaft length (25 cm) and the ability to rotate

A microwave surgical instrument (MSI) (Acrosurg.®, Nikkiso Co Ltd, Tokyo, Japan; Fig. 1a) is a novel surgical energy instrument that is based on microwave technology and was approved by the Japanese Pharmaceuticals and Medical Devices Agency in 2016 [1]. Microwave radiations have a frequency of 2450 MHz, which is the same wavelength as that in a microwave oven, and can heat body tissues through vibrating water molecules, creating a uniform coagulation layer [2]. Rather than producing carbonization, microwaves cause dehydration and fixation and have strong hemostatic and sealing ability. A previous study found that the burst pressure of vessel sealing devices is similar to that of other sealing devices [2, 3]. The temperature of tissues subjected to microwaves does not increase above 100℃; therefore, the spread of heat to the surrounding tissues can be avoided. The MSI allows a series of maneuvers, including fine tissue dissection, coagulation, hemostasis, and vessel sealing, by clinching with a scissor-type blade while emitting microwave energy (Fig. 1b, c) [3, 4]. Therefore, the MSI has been rapidly adopted in the gastrointestinal surgery field [1, 5].

We recently demonstrated the usefulness of this MSI in lung segmentectomy after verification of its safety in animal experiments, wherein it allowed excellent sealing in lung parenchyma dissection [6]. In the present study, we used this MSI in various lung resection situations, including vessel sealing, and in cases wherein the use of an automatic suturing instrument (ASI) was difficult. Here, we describe our experience of the clinical utility of this novel device in three patients (cases 1–3).

## Clinical experience

In all the cases, we performed complete video-assisted thoracoscopic surgery using a three-dimensional system (Endoeye Flex [LTF-190-10-3D]; Olympus, Tokyo, Japan).

### Wedge resection for metastatic lung cancer located near the pulmonary hilum (case 1)

**Fig. 2:**
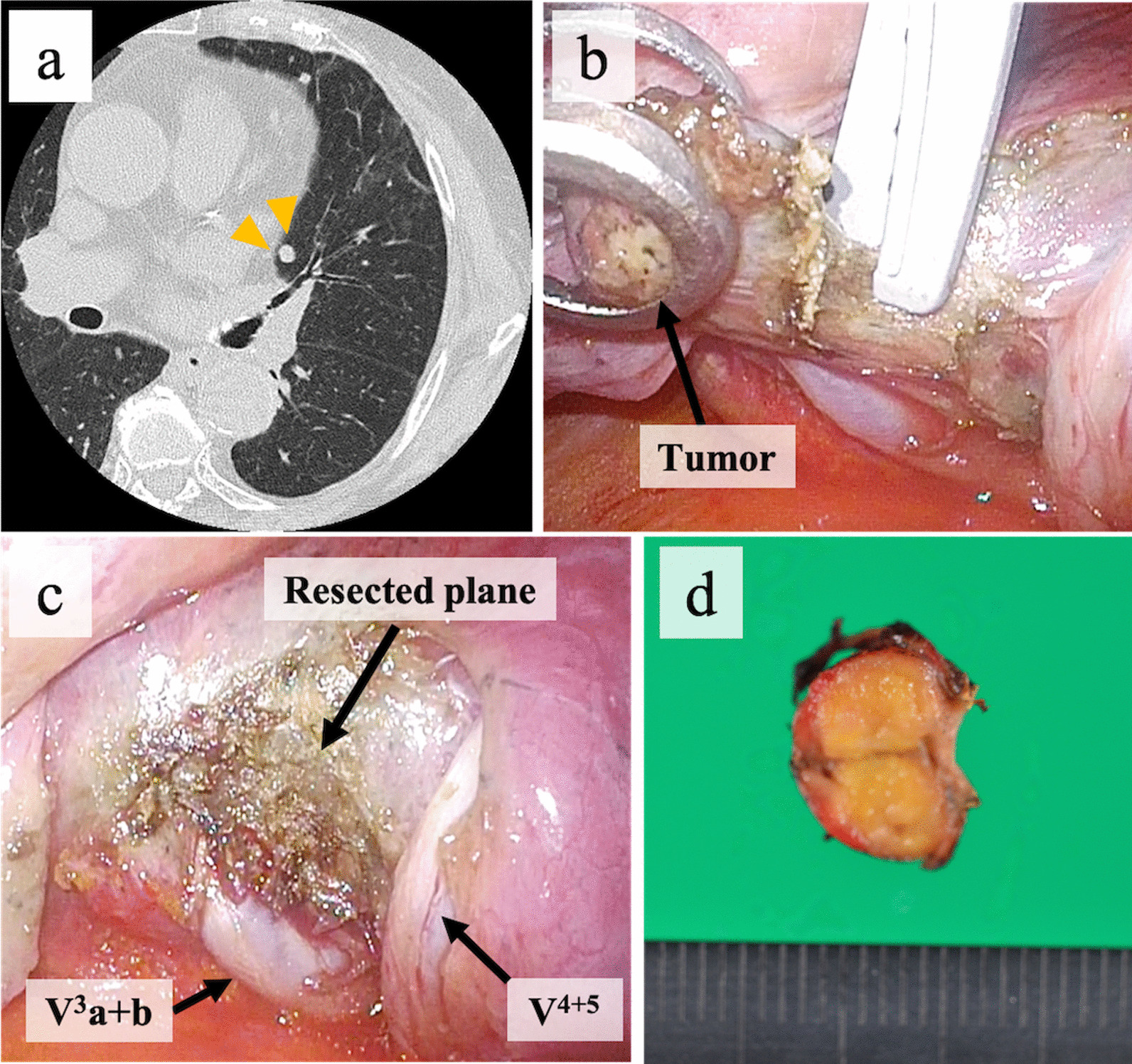
Wedge resection for a metastatic lung cancer located close to the pulmonary hilum. (**a)** A high-resolution computed tomography scan showing a pulmonary nodule located close to the pulmonary hilum (yellow arrowheads). (**b)** The pulmonary nodule is grasped by forceps, and the lung parenchyma is dissected sharply using the microwave surgical instrument. (**c)** Thick heat-denatured lung tissues overlie the resected surface with adequate sealing. (**d)** The resected surgical specimen including the tumor

An 87-year-old woman with suspected lung metastasis from thyroid cancer underwent wedge resection [see Additional file 1]. Computed tomography revealed a 6-mm pulmonary nodule located close to the pulmonary hilum in the left upper lobe (Fig. 2a). A pulmonary nodule was identified between the base of V^3^a + b and V^4+5^. An ASI could not be inserted; the pulmonary nodule was grasped by forceps, and the lung parenchyma was dissected sharply using the MSI with confirmation of a safe surgical margin (Fig. 2b). The resected parenchymal surface was sealed adequately, with no air leak observed on a water sealing test with a pressure of 20 cmH_2_O (Fig. 2c). The chest tube was removed on postoperative day 2, and the patient was discharged without any complications. Pathologically, the tumor was diagnosed to be a lung metastasis from thyroid cancer (Fig. 2d).

### Use of the MSI to divide the intersubsegmental plane in complex subsegmentectomy (case 2)

**Fig. 3:**
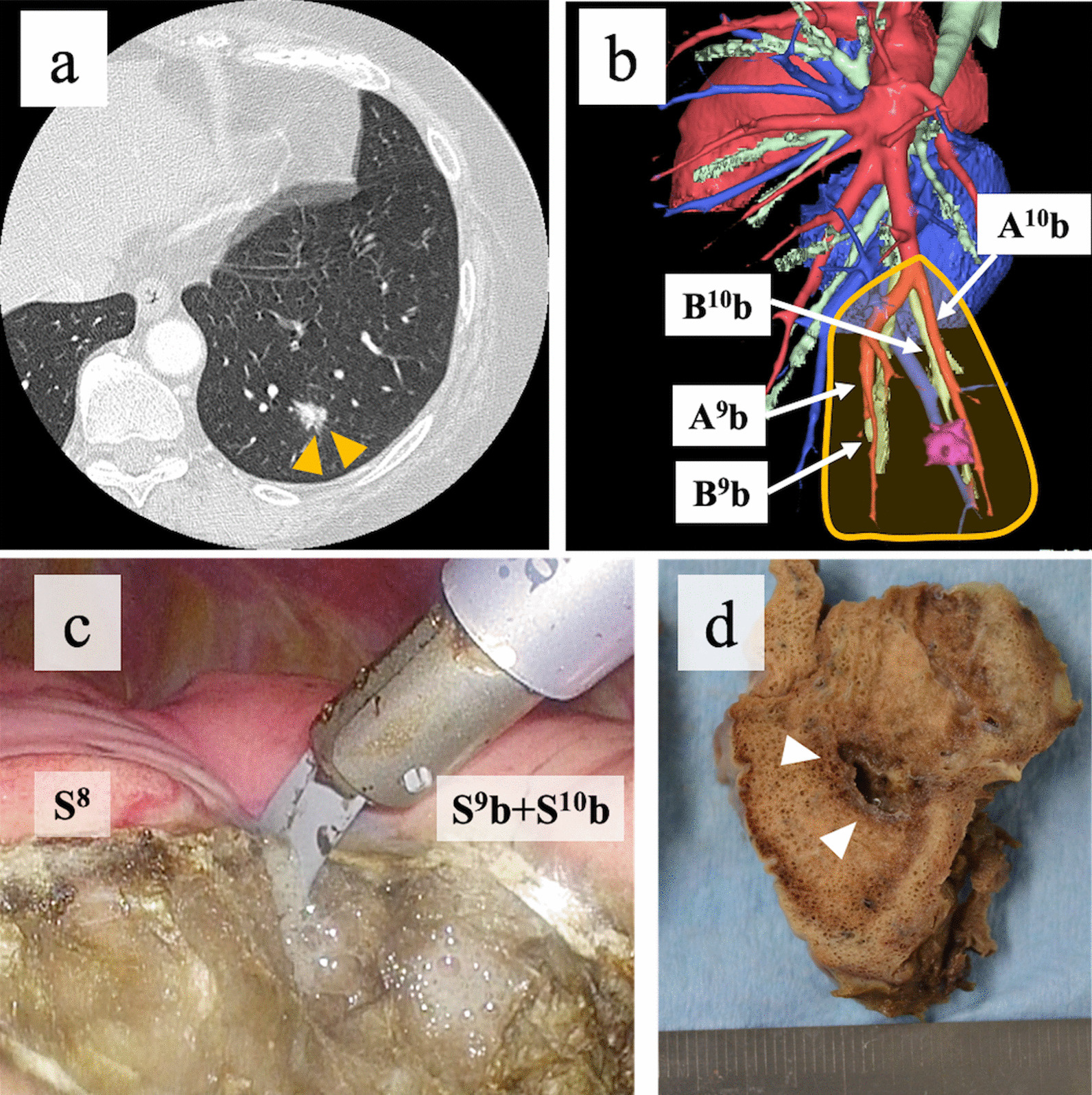
Complex subsegmentectomy (the left S^9^b + S^10^b segment) for an indeterminate pulmonary nodule beneath the pleural surface. (**a)** High-resolution CT and (**b)** three-dimensional CT images obtained using the Synapse Vincent imaging software program (Fujifilm Medical, Tokyo, Japan) showing a pulmonary nodule in the left S^9^b + S^10^b segment. The yellow box indicates the area to be resected. (**c)** The lung parenchyma is dissected along the intersubsegmental plane using a microwave surgical instrument. (**d)** The resected surgical specimen showing a tumor (white arrowheads) with an adequate margin. S^9^b, lateral basal subsegment basal; S^10^b, posterior basal subsegment basal; CT, computed tomography

A 62-year-old woman with an indeterminate nodule at a position deeper than the pleural surface underwent S^9^b (lateral basal subsegment basal) and S^10^b (posterior basal subsegment basal) subsegmentectomy [see Additional file 2]. Computed tomography revealed a 10-mm nodule at a position deeper than the pleural surface in the left S^9^b + ^10^b (Fig. 3a, b). After suspecting a primary lung cancer or lung metastasis of advanced ovarian cancer, we performed subsegmentectomy to secure an adequate surgical margin. The lung parenchyma was dissected along V^9+10^ from the pulmonary hilum using the MSI via a dorsal approach, resulting in exposure of the A^9^b + ^10^b and B^9^b + ^10^b segments. The S^9^b + S^10^b segment was inflated by jet ventilation, and the B^9^b + ^10^b segment was dissected. The inflation–deflation line was unclear; therefore, the segmental line was decided based on the surrounding structures and the margin from the tumor. The lung parenchyma was dissected along the marking of the segmental line using the MSI, and the S^9^b + S^10^b segment was excised (Fig. 3c). Only a minor air leak was observed on the water sealing test at a pressure of 20 cmH_2_O. Finally, a polyglycolic acid sheet and fibrin glue were patched onto the intersegmental plane. The chest tube was removed on postoperative day 2, and the patient was discharged without any complications. Pathologically, the lung tumor was diagnosed to be a pathological stage IA1 papillary adenocarcinoma (Fig. 3d).

### Use of the MSI to divide incomplete interlobular fissure (case 3)

**Fig. 4:**
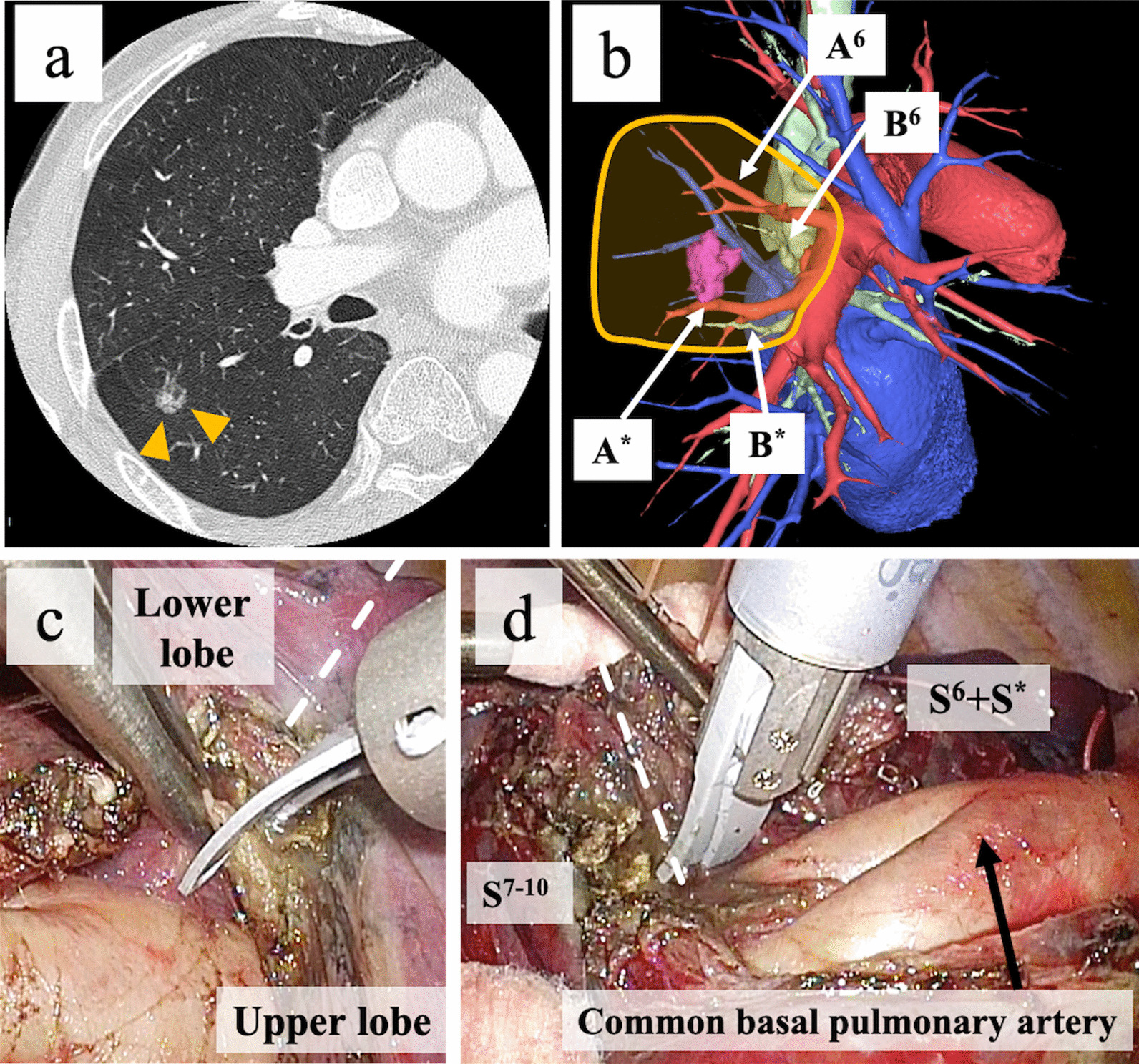
Segmentectomy (the S^6^ + S* segment) for lung cancer with incomplete lobulation. (**a)** High-resolution CT and (**b)** three-dimensional CT images showing a pulmonary nodule in the right S^6^ + S* segment. The yellow box indicates the area to be resected. (**c)** The interlobar lung parenchyma with incomplete lobulation between the right upper and lower lobes (white dotted line) is dissected using the microwave surgical instrument toward the second carina. (**d)** The S^6^ + S* segment including the tumor emerges sufficiently from the pulmonary hilum and is subsequently sectioned off between the intersegmental plane (white dotted line) by an ASI. S^6^, superior segment; S*, subsuperior segment; CT, computed tomography

A 75-year-old man with a primary lung cancer underwent S^6^ (superior segment) and S* (subsuperior segment) segmentectomy [see Additional file 3]. Computed tomography revealed a 15-mm nodule located at the border of the right S^6^ and S* segments with an incomplete interlobar fissure (Fig. 4a, b). The common basal pulmonary artery was barely identified in the incomplete interlobar fissure between the right middle and lower lobes, after which the lung parenchyma was divided along the pulmonary artery using the MSI. As the procedure exposed the entire pulmonary artery in the interlobar area, lung parenchyma dissection was advanced toward the second carina to facilitate tunneling beneath the interlobar lung parenchyma between the right upper and lower lobes (Fig. 4c), followed by separation of the interlobar lung parenchyma with an ASI. Then, the V^6^ and B^6^ segments were dissected on the dorsal side, and the A^6^, A*, and B* segments were dissected on the interlobar side. The S^6^ + S* segment involving the tumor emerged sufficiently from the pulmonary hilum and was subsequently sectioned off between the intersegmental plane by the ASI (Fig. 4d). No air leak was observed by the water sealing test at a pressure of 20 cmH_2_O. The chest tube was removed on postoperative day 2, and the patient was discharged without any complications. Pathologically, the lung tumor was diagnosed to be a pathological stage IA1 acinar adenocarcinoma.

## Discussion

The MSI is very useful in various lung resection cases, especially when an ASI is difficult to use. Fine dissection of the lung parenchyma with the MSI contributes to the smooth progression of surgery in such cases.

An ASI is used for lung parenchyma dissection, including wedge resection, segmentectomy, or lobectomy. Although an ASI has the advantage of preventing air leak when dissecting the lung parenchyma, it is sometimes unavailable or difficult to use in cases wherein tumors are located close to the hilum or when incomplete interlobar fissure separation, complex segmentectomy, or subsegmentectomy is needed, as in the above-mentioned cases. A device that can cut the lung parenchyma sharply without any air leak in such situations would be accepted by thoracic surgeons.

Similar to other energy devices, such as the LigaSure [7], the MSI is capable of sealing and dissecting a vessel with a diameter of ≤ 5 mm. The strong sealing ability of the MSI indicates that it can also be used for dissection of the lung parenchyma. The Acrosurg.® MSI is designed to sharply dissect tissues using a ceramic blade while simultaneously coagulating and sealing the tissue. Although it is sometimes difficult to determine whether the lung parenchyma could be cut after sufficient coagulation, we determine that closure occurs when the water completely evaporates and when the color of the dissection plane changes to khaki. From a pathological point of view, strong thermal denaturation has been observed in the intersegmental plane when this MSI is used in lung segmentectomy [6]. Furthermore, no surgical smoke or mist is generated, and little cavitation is noted in the surrounding tissue because the microwaves heat the tissue through vibrating water molecules alone. The MSI is a safe and effective technology for use in general thoracic surgery. It was particularly useful in the above-mentioned patients, who required fine dissection of the lung parenchyma during resection of a pulmonary nodule located close to the pulmonary hilum, who had a lung with an incomplete interlobar fissure, or who underwent complex segmentectomy/subsegmentectomy. In the case of extensive parenchyma resection with the MSI, such as a complex segmentectomy, minor air leaks may occur because the area to be actively cut increases, but there is no problem with a combination of polyglycolic acid sheet and fibrin glue patch, as noted in case 2. Even in segmentectomy/subsegmentectomy with an ASI, as noted in case 3, dissection of the lung parenchyma at the hilum allows smooth insertion of an ASI in the correct direction, especially in endoscopic surgery. The MSI is also useful for dissection of lymph nodes, pulmonary vessels, and adhesions and for repair of pulmonary fistulae by coagulation.

The main drawbacks of the MSI are its short shaft length of 17 cm and its inability to rotate, indicating that it is not ideal for video-assisted thoracoscopic surgery. However, a more recent device (Acrosurg.® Revo, Nikkiso; Fig. 1d) with a longer shaft length (25, 35, or 45 cm) and an ability to rotate has been developed to allow more precision in endoscopic surgery. The results of its usefulness are expected in the future.

## Conclusion

The MSI allows fine tissue dissection and excellent tissue sealing and, thus, may be an ideal device for use in general thoracic surgery. This device can assist thoracic surgeons in various lung resection situations. Further prospective comparative research is needed to determine the long-term outcomes of the use of this MSI, including the Acrosurg.® Revo MSI, in thoracic surgery.

## Supplementary Information

Additional file 1. Wedge resection for metastatic lung cancer located near the pulmonary hilum.

Additional file 2. The use of the MSI to divide the intersubsegmental plane in complex subsegmentectomy.

Additional file 3. The use of the MSI to divide incomplete interlobular fissure.

## Data Availability

The datasets used during the current study are available from the corresponding author on reasonable request.

## Abbreviations

ASI: Automatic suturing instrument
CT: Computed tomography
MSI: Microwave surgical instrument
